# Five-Year Survival After Transcatheter Versus Surgical Aortic Valve Replacement in Patients with Severe Aortic Valve Stenosis—Do We Choose the Right Treatment for Each Patient? A Propensity Score Matched Analysis

**DOI:** 10.3390/jpm15080391

**Published:** 2025-08-20

**Authors:** George Samanidis, Antonios Roussakis, Sotirios Katsaridis, Efthymia Liaretidou, Eirini Kefalidi, Areti Falara, Ilias Georgios Koziakas, Ioannis Nenekidis, Ilias Kosmas, Evangelos Leontiadis, Vassilios Voudris, Ioannis Iakovou, Konstantinos Perreas

**Affiliations:** 1 Department of Cardiac Surgery, Onassis Hospital, 17674 Athens, Greece; antonisroussakis@yahoo.gr (A.R.); katsaridisdoc@yahoo.gr (S.K.); euthliar@gmail.com (E.L.); eirkef.ek@gmail.com (E.K.); jonenek@yahoo.com (I.N.); perreas@doctors.org.uk (K.P.); 2 Department of Anesthesiology, Onassis Hospital, 17674 Athens, Greece; areti_f@hotmail.com (A.F.); iliaskoziakas@outlook.com (I.G.K.); 3 Department of Interventional Cardiology, Onassis Hospital, 17674 Athens, Greece; ekosmas@gmail.com (I.K.); evanleont@gmail.com (E.L.); v.voudris@onasseio.gr (V.V.); ioannis.iakovou@gmail.com (I.I.)

**Keywords:** aortic valve stenosis, surgical aortic valve replacement, transcatheter aortic valve implantation, low–medium risk patients, personalized treatment

## Abstract

**Background and Objectives:** The treatment of choice for aortic valve stenosis in patients with low and intermediate risk is still debated. In this study, we compared the outcomes of low-to-intermediate surgical risk patients who underwent surgical versus transcatheter aortic valve replacement for severe aortic valve stenosis (AS). **Methods:** Between 2015 and 2019, 326 consecutive patients with severe AS underwent transcatheter aortic valve implantation (TAVI), while 341 patients underwent surgical aortic valve replacement (SAVR). The two populations were propensity score matched by age, gender and Euroscore II. The survival rate of patients during median 5-year follow-up between SAVR and TAVI patients was evaluated. **Results:** After propensity score matching, 94 pairs of patients were compared and the mean standard deviation age of patients, sex (female) and Euroscore II were 77.5 (6.6) versus 76.6 (6.5) years, 51.1% versus 51.1% and 3.3 (1.88)% versus 3.0 (1,84)%, respectively. Permanent pacemaker implantation was higher in transcatheter group (21.3% versus 1.1%, *p* < 0.001). No difference in length of ICU and in-hospital stay was observed, *p* = 0.08 and *p* = 0.12, respectively. During follow-up the presence of more than moderate insufficiency of the prosthetic valve postoperatively was significantly less frequent in the surgical versus transcatheter (0% versus 14.3%). Survival rates over 1, 3 and 5 years did not differ in surgical versus transcatheter group (93.6%, 81.9% and 62.8% versus 86.2%, 69.1% and 59.6%, respectively (*p* = 0.16)). **Conclusions:** Short- and long-term survival rates were similar in patients who underwent transcatheter versus surgical aortic valve replacement, whereas SAVR showed superior results concerning the postoperative detection of residual regurgitation and need for PPM. It is extremely important to personalize the choice of treatment according to patients’ age, clinical status and life expectancy.

## 1. Introduction

Severe aortic valve stenosis (AS) is the most frequent valvular pathology. Over the last decade, transcatheter aortic valve implantation (TAVI) established itself as a viable non-inferior, or even superior, treatment that is a less invasive and more reliable alternative modality to surgical aortic valve replacement (SAVR) in low- and intermediate-risk elder patients [1,2,3].

In this study, we present propensity matched outcomes of a heart valve tertiary center in patients with severe AS who underwent TAVI versus SAVR.

## 2. Patients and Methods

### 2.1. Study Population

Between 2015 and 2019, 326 consecutive patients with severe AS underwent TAVI procedure, while the SAVR group included 341 consecutive patients who underwent isolated aortic valve replacement (with full sternotomy or minimally invasive techniques) from a single tertiary Cardiac Surgery Department. The two populations were propensity score matched for age, sex and Euroscore II. After propensity score matching, 98 pairs of patients were created for comparison. Long-term survival during median 5-year follow-up between SAVR and TAVI patients was evaluated. The type of intervention for aortic valve replacement was based on the decision of the hospital heart team.

### 2.2. Ethical Statement

The study has been performed in accordance with the ethical standards of the Declaration of Helsinki as revised in 2013. Also, the study was approved by our hospital Institutional Review Board (804/22.4.24). Receiving informed consent from patients was waived due to the nature of study (retrospective study).

### 2.3. Statistical Analysis

Normally distributed variables are expressed as mean ± standard deviation (SD), while variables with skewed distribution are expressed as median and interquartile range (IQR). Qualitative variables were expressed as absolute and relative frequencies. The normality assumption was evaluated using Kolmogorov–Smirnov test. If the normality assumption was satisfied for the comparison of means between two groups, Student’s *t*-test was used. Mann–Whitney test was used for the comparison of continuous variables between two groups when the distribution was not normal. For the comparisons of proportions chi-square tests and Fisher’s exact tests were used. Propensity score matching was performed to generate a study cohort of matched patients undergoing conventional TAVI and SAVR based on sex, age and Euroscore II. Propensity scores were estimated using logistic regression models with performance of surgery method as the dependent variable. Propensity score matched cohort was constructed by nearest neighbor matching of one patient undergoing TAVI to one patient undergoing SAVR. Two kinds of comparisons were conducted: one included all patients and the second included TAVI and SAVR groups after the propensity score matching. Long-term survival was evaluated by Kaplan–Meier analysis. Log-rank test was used for comparing survival rate between SAVR and TAVI group. Statistical significance was set at 0.05 and analyses were conducted using SPSS statistical software (version 25.0).

## 3. Results

The study sample consisted of 326 patients in the TAVI group and 341 patients in the SAVR. In total, 326 consecutive patients with severe AS underwent TAVI procedure through various approaches but mainly through trans-femoral (Ν = 280 [85.9%]), trans-subclavian (Ν = 17 [5.2%]) or trans-apical (N = 14 [4.3%]) approach. Deferent implantation devices for TAVI were used [Medtronic Evolut Pro = 48 (14.7%), Medtronic Evolut R = 240 (73.6%), Medtronic Core Valve = 18 (5.5%), Edwards Sapien = 17 (5.2%) and Edwards Sapien S3 = 3 (0.9%)], with Evolut R of Medtronic being the most popular (Ν = 240, [73.6%]). In the SAVR group (N = 341), 285 (82.6%), patients were treated via full sternotomy, while 56 (16.4%) were treated with mini sternotomy. Devices for SAVR were bioprosthetic valve [ST. Jude TRIFECTA = 146 (42.8%), Labcore = 30 (8.8%), Medtronic Avalus = 15 (4.4%), Sorin Mitroflow = 48 (14.1%), PERCEVAL S = 6 (1.8%), Magna Ease = 3 (0.9%)] and mechanical valve [ATS = 35 (10.3%), ST. Jude = 2 (0.6%), Sorin Bicarbone = 44 (12.9%), On-X = 12 (3.5%)]. After propensity score matching, 94 patients with TAVI were matched with 94 patients with SAVR for sex, age and Euroscore II. Table 1 shows basic characteristics for TAVI and SAVR before and after matching.

Preoperative characteristics for TAVI and SAVR groups before and after propensity score matching are shown in Table 2. Before matching, patients of SAVR group had more weight, lower size, lower proportion of pulmonary artery hypertension (PAH) and peripheral artery disease (PAD). After matching, SAVR group had lower size and lower proportion of PAH and PAD. Also, differences in left ventricular ejection fraction (LVEF) levels were found before and after matching. The percentage of carotid disease was significantly lower, while the percentage of hyperlipidemia was significantly higher in the SAVR group before and after matching.

Table 3 shows postoperative complications for the two groups before and after propensity score matching. Proportion of Valve Academic Research Consortium-2 (VARC) was significantly higher in SAVR group, while permanent pacemaker (PPM) implantation rate was less frequent in SAVR group both before and after matching. Before matching, other complications were more frequent in SAVR group but after matching this difference did not reach statistical significance.

**Figure 1:**
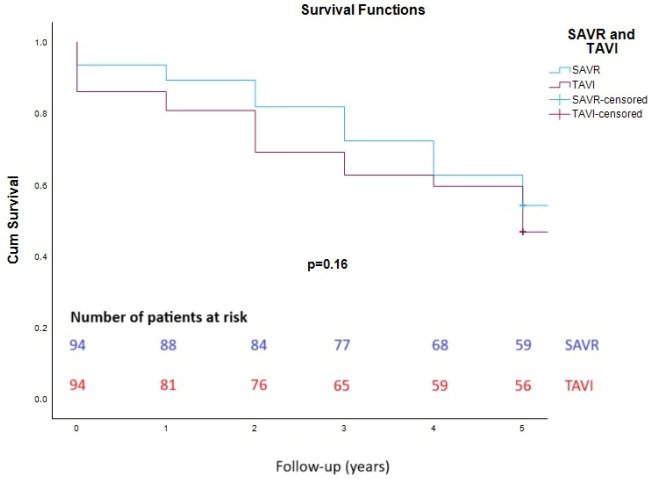
Kaplan–Meier survival analysis. SAVR = surgical aortic valve replacement; TAVI = transcatheter aortic valve implantation. Log-rank test *p* = 0.16.

Thirty-day mortality rates were similar for the two groups, both before and after matching (Table 4). Furthermore, prosthetic aortic valve regurgitation (AR) postoperatively was lower for SAVR group. The lengths of both in-hospital and ICU stays were lower for the SAVR group before matching (Table 5). After matching, there were no significant differences in length of in-hospital and ICU stay between the two groups. There was no statistically significant difference in survival rates 1, 3 and 5 years after intervention between the two groups (SAVR versus TAVI) (93.6%, 81.9% and 62.8% versus 86.2%, 69.1% and 59.6%, respectively (*p* = 0.16)) (Figure 1). There were no patients lost to follow-up.

## 4. Discussion

This is a real-world, single, high-volume, tertiary valve center propensity matched comparison study of low–medium-risk patients receiving either TAVI or SAVR. We found similar all-cause mortality between TAVI and SAVR in both the general and PSM-adjusted samples.

In terms of other periprocedural complications, we found higher bleeding rates in the SAVR group compared to TAVI. In the SAVR group, about half of the patients (48.7% in the total sample and 46.8% in the simulated sample) required transfusion with more than one unit of concentrated red blood cells versus approximately 15% of patients in the TAVI group. From the surgical point of view, a reduction in the need for transfusion can perhaps be achieved by adopting minimally invasive techniques and using blood collection devices from the surgical field and transfusing it directly to the patient intraoperatively (cell-saver devices). There was not enough power to draw conclusions about this due to the small number of minimal invasive cardiac surgery (MICS) cases included. Since 2019, our practice has shifted to MICS-only techniques for SAVR.

Regarding the duration of hospitalization and the time needed in the intensive care unit, they are not fully compatible with the results of other studies. We found that patients undergoing TAVI stay statistically significantly longer in the hospital and ICU after the procedure compared to patients undergoing SAVR surgery. However, this difference ceases to be significant in the cross-matched sample of 196 patients. There remains only a statistically marginal trend for shorter hospital stays in favor of SAVR patients (*p* = 0.08).

Following the trend for extension of the TAVI criteria to younger and lower-risk patients, several randomized controlled trials (RCTs), as well as registry and observational studies comparing the two therapeutic methods, have been published [1,2,3,4]. On a parallel trend, surgical intervention shifts to less invasive strategies as well as more durable and valve-in-valve friendly bio prosthetic valves [5]. In our center, the treatment of patients with isolated severe aortic valve stenosis, patients aged >75 years old and patients <75 years old with severe comorbidities by TAVI is based on local heart team decisions. Also, the treatment of patients with TAVI procedure needs approval by each hospital committee and national committee. In our country there is a mandatory process. Thus, in the time frame of the study there remained a preponderance of elder and higher-risk patients receiving TAVI, as shown in the initial study population prior to the matching process. However, the expected time lag in the shifting paradigm allowed for an overlap of similar patients in the two therapeutic arms. These resulted in patients with mean age of 77 versus 78 years old and mean Euroscore II of 3.3 versus 3 between SAVR and TAVI. After matching, TAVI patients were more likely to have concomitant carotid disease and PAH and less likely to have diabetes mellitus, renal failure and hyperlipidemia. However, unlike most RCTs on the matter, and per study design, there were no patients with significant coronary artery disease (CAD) requiring revascularization in either subgroup, making this, we believe, a more balanced comparison. Short-term results showed similar peri-op mortality and non-statistically significant survival difference at median 5-year follow-up between the two groups (*p* < 0.16). Furthermore, the lower rate of PPM implantation for the SAVR was noted, very much in accordance with the NOTION and PARNTER 3 RCTs results as well as registry studies [6,7]. It is, however, clear that more time and possibly larger numbers are required to delineate the pros and cons of each method in comparison. Furthermore, longer-term data and better randomization studies are required for younger patients. Similar survival for patients aged >75 may not be safely adopted for low- and intermediate-risk younger patients, as the numbers are greatly affected by the natural survival curve of the elder patients. In another study, most TAVI patients were dead by 10 years, probably due to their age at the time of implantation obscuring the real valve durability and long-term survival [8].

In a recent meta-analysis of RCTs and propensity matched studies, TAVI versus SAVR was inconclusive about all-cause mortality at 4–5 years in patients with low or intermediate surgical risk [9,10]. Meta-regression time demonstrated a trend to decrease 1-year mortality followed by an increasing trend at 3–5 years in the risk of all-cause mortality after TAVI compared with SAVR. TAVI was generally associated with a higher risk of moderate or severe aortic regurgitation, moderate or severe paravalvular regurgitation, and pacemaker placement [9]. In a Japanese meta-analysis of 5498 patients with 5-year follow-up, there was a trend towards improved survival for SAVR in low-risk patients [11]. However, the study did not include the recently published long-term PARTNER 3 results. Our study suffers from weaknesses inherent to most propensity matched studies. Therefore the two groups were not similar in all characteristics. The number of patients included is small and could possibly not have allowed for the recognition of other differences that do exist.

The results of the currently seminal controlled randomized trials, and the consequent generalization to all real-world patient characteristics, have been questioned by specific groups, due to systematic exclusion from clinical trials of patients with certain high-risk comorbidities (e.g., dialysis, recent stroke [<6 months ago], very low left ventricular ejection fraction (<20%), functionally bicuspid aortic valve, etc. [1,2,3,4,6,7,8]. In contrast, a homogenized sample study that included 5997 intermediate-risk patients who underwent TAVI versus SAVR as part of the GARY database (German Aortic Valve Registry), showed that treatment with TAVI had similar in-hospital mortality [12]. Contrary to the results of the GARY study, we did not observe an increased risk of mortality in TAVI patients among patients at intermediate risk.

TAVI, as a procedure, is established as a standard treatment of choice in specific groups of patients with severe AS: for patients >75 years old, based on recent ESC guidelines (2021) and AHA guidelines (2020) regarding age of patients, the decision should be made after heart team procedure [13,14]. On the other hand, the comparison of mid- and long-term survival of patients who underwent SAVR and TAVI with low and intermediate risk is debated. TAVI is chosen as an appropriate treatment for elderly patients >75 years old with acceptable outcomes and SAVR seems to offer better outcomes in younger patients <75 and <65 years old. Although mild-to-moderate paravalvular leak and PPM insertion are very rarely observed in patients after SAVR, the same post-procedure TAVI complications are more frequently observed after TAVI. While, in older patients (>75 years old), post-TAVI these complications are due to the age of patients, comorbidities and expectation of life can be treated conservatively, in the younger patients <75 and <65 years old, these complications may affect the quality of life (readmission in hospital, possible infection after PPM insertion, reintervention, prolonged stay in ICU and in-hospital stay). Also in our study, in practice, we compared two groups with patients with low risk with mean ES II < 4%. Many studies addressed and discussed the survival rate after 2 or 3 years. Few patients with 5-year survival rate are presented. We believe that our study adds to updates about outcomes after TAVI vs. SAVR in patients with low to intermediate risk. On the other hand, our study confirms that post-TAVI PVL and PPM insertion are computable adverse events, while after SAVR the rate of these events is lower. The positive conclusion is that survival rate is the same between TAVI and SAVR about all-cause mortality.

In conclusion, it should be noted that in this study we try to compare subgroups of patients treated worldwide with both techniques (age of patients 75–80 years old and low–medium-risk patients). Furthermore, longer term results, we believe, always offer further insight as to the potential advantages of each method.

## 5. Study Limitations

This study is a single-center experience. The number of patients in comparison following cross-matching in this study is small to allow generalization. ICU protocols seem to differ between the two therapeutic cohorts. Retrospective propensity score matching is prone to the inherent weaknesses of such comparisons. Judicious decision-making of a well-organized heart team may also be obscuring real differences (if present) of the two methods. Both TAVI and SAVR valves, as well as the structured minimization of invasiveness of both procedures, continue to evolve, therefore driving a continuum of improvement in the results.

## 6. Conclusions

In conclusion, this is a propensity matched study comparing short-, mid- and long-term results between SAVR and TAVI in low–intermediate-risk patients treated in a tertiary heart surgery center. Our results showed that short- and long-term all-cause mortality rates were similar in patients who underwent SAVR versus TAVI, while postoperative PPM implantation and prosthetic valve regurgitation rates were higher in TAVI patients. A careful choice of treatment should be made, especially in low-to-intermediate risk patients, since SAVR, which has been the gold standard approach over the last 50 years, remains comparable if not superior to TAVI.

## Figures and Tables

**Table 1:** Sex, age and Euroscore II before and after propensity score matching in TAVI and SAVR patients. ^+^ Pearson’s chi-square test; ^‡^ Student’s *t*-test; N = number; SD = standard deviation; TAVI = transcatheter aortic valve implantation; SAVR = surgical aortic valve replacement.

	Total Sample (N = 667)			Propensity Score Matched Sample (N = 188)		
	TAVI (N = 326)	SAVR (N = 341)	*p*	TAVI (N = 94)	SAVR (N = 94)	*p*
Age, years old, (SD)	81.0 (6.5)	70.6 (10.0)	<0.001 ^‡^	77.5 (6.6)	76.6 (6.5)	0.35 ^‡^
Sex, N (%)						
Female	146 (44.8)	141 (41.3)	0.37 ^+^	48 (51.1)	48 (51.1)	1.00 ^+^
Male	180 (55.2)	200 (58.7)		46 (48.9)	46 (48.9)	
Euroscore II, %, (SD)	6.48 (4.86)	1.68 (1.29)	<0.001 ^‡^	3.31 (1.88)	3.0 (1.84)	0.25 ^‡^

**Table 2:** Preoperative characteristics for TAVI and SAVR groups before and after propensity score matching. ^+^ Pearson’s chi-square test; ^++^ Fisher’s exact test; ^‡^ Student’s *t*-test; N or n = number; SD = standard deviation; TAVI = transcatheter aortic valve implantation; SAVR = surgical aortic valve replacement; LVEF = left ventricular ejection fraction.

	Total Sample (N = 667)			Propensity Score Matched Sample (N = 188)		
	TAVI (N = 326)	SAVR (N = 341)	*p*	TAVI (N = 94)	SAVR (N = 94)	*p*
	N (%)	N (%)		N (%)	N (%)	
Weight, kgr, (SD)	73.3 (13.6)	80.7 (16.7)	<0.001 ^‡^	75.7 (15.7)	76.6 (17)	0.73 ^‡^
Prosthetic aortic valve size, mm, (SD)	27.5 (3.0)	21.4 (5.6)	<0.001 ^‡^	27.1 (2.9)	20.9 (2.1)	<0.001 ^‡^
Preoperative LVEF, n (%)						
>50%	234 (71.7)	267 (78.3)	0.01 ^+^	85 (90.4)	67 (71.3)	0.004 ^++^
30–49%	77 (23.6)	70 (20.5)		6 (6.4)	25 (26.6)	
<29%	15 (4.7)	4 (1.2)		3 (3.2)	2 (2.1)	
Pulmonary artery hypertension, n (%)	127 (39.0)	26 (7.6)	<0.001 ^+^	28 (29.8)	15 (16.0)	0.02 ^+^
Peripheral artery disease, n (%)	88 (27.0)	15 (4.4)	<0.001 ^+^	17 (18.1)	6 (6.4)	0.01 ^+^
Chronic obstructive pulmonary disease, n (%)	92 (28.2)	89 (26.1)	0.53 ^+^	25 (26.6)	26 (27.7)	0.87 ^+^
Preoperative renal failure, n (%)	38 (11.7)	38 (11.1)	0.83 ^+^	7 (7.4)	15 (16)	0.11 ^+^
Diabetes mellitus, n (%)	64 (19.6)	77 (22.6)	0.35 ^+^	16 (17.0)	25 (26.6)	0.11 ^+^
Carotid disease (>70%), n (%)	81 (25.2)	6 (1.8)	<0.001 ^+^	16 (17.0)	2 (2.1)	0.001 ^+^
Hypertension, n (%)	226 (70.4)	253 (74.2)	0.27 ^+^	58 (61.7)	69 (73.4)	0.08 ^+^
Hyperlipidemia, n (%)	138 (43.0)	191 (56.0)	0.001 ^+^	33 (35.1)	64 (68.1)	<0.001 ^+^

**Table 3:** Complications for TAVI and SAVR groups before and after propensity score matching. ^+^ Pearson’s chi-square test; ^++^ Fisher’s exact test; ^‡^ Not computed due to no distribution; N or n = number; TAVI = transcatheter aortic valve implantation; SAVR = surgical aortic valve replacement; PPM = permanent pacemaker; VARC = Valve Academic Research Consortium-2.

	Total Sample (N = 667)			Propensity Score Matched Sample (N = 188)		
	TAVI (N = 326)	SAVR (N = 341)	*p*	TAVI (N = 94)	SAVR (N = 94)	*p*
	N (%)	N (%)		N (%)	N (%)	
Permanent cerebrovascular accident, n (%)	1 (0.3)	1 (0.3)	1.00 ^++^	0 (0.0)	0 (0.0)	- ^‡^
Transient ischemic attack, n (%)	7 (2.1)	2 (0.6)	0.10 ^++^	0 (0.0)	0 (0.0)	- ^‡^
Major bleeding (VARC-2), n (%)	54 (16.6)	166 (48.7)	<0.001 ^+^	14 (14.9)	44 (46.8)	<0.001 ^+^
Surgical intervention, n (%)	14 (4.3)	N/A	- ^‡^	3 (3.2)	N/A	- ^‡^
PPM implantation, n (%)	61 (18.7)	7 (2.1)	<0.001 ^+^	20 (21.3)	1 (1.1)	<0.001 ^+^
Other complications	95 (29.1)	144 (42.2)	<0.001 ^+^	23 (24.5)	34 (36.2)	0.08 ^+^

**Table 4:** Mortality and other outcomes for TAVI and SAVR groups before and after propensity score matching. ^+^ Pearson’s chi-square test; ^++^ Fisher’s exact test. TAVI = transcatheter aortic valve implantation; SAVR = surgical aortic valve replacement.

	Total Sample (N = 667)			Propensity Score Matched Sample (N = 188)		
	TAVI (N = 326)	SAVR (N = 341)	*p*	TAVI (N = 94)	SAVR (N = 94)	*p*
	N (%)	N (%)		N (%)	N (%)	
30-day mortality, n (%)	7 (2.1)	4 (1.2)	0.32 ^+^	0 (0)	2(2.1)	0.49 ^++^
Degree of postoperative prosthetic aortic valve regurgitation, n (%)						
0	49 (24.9)	338 (99.1)	<0.001 ^++^	14 (28.6)	91 (96.8)	<0.001 ^++^
1	120 (60.9)	3 (0.9)		28 (57.1)	3 (3.2)	
2	27 (13.7)	0 (0.0)		7 (14.3)	0 (0.0)	
3	1 (0.5)	0 (0.0)		0 (0.0)	0 (0.0)	
4	0 (0.0)	0 (0.0)		0 (0.0)	0 (0.0)	
Degree of postoperative prosthetic aortic valve regurgitation, n (%)						
0–1	169 (85.9)	341 (100.0)	<0.001 ^+^	42 (85.7)	94 (100.0)	<0.001 ^+^
2–4	28 (14.1)	0 (0.0)		7 (14.3)	0 (0.0)	

**Table 5:** Length of hospital and ICU stay for the two study groups before and after propensity score matching. ^‡‡^ Mann–Whitney test; TAVI = transcatheter aortic valve implantation; SAVR = surgical aortic valve replacement.

	Total Sample (N = 667)			Propensity Score Matched Sample (N = 188)		
	TAVI (N = 326)	SAVR (N = 341)	*p* ^‡‡^	TAVI (N = 94)	SAVR (N = 94)	*p* ^‡‡^
Length of ICU stay, days, (IQR)	2 (2–3)	1.3 (1.3–2.0)	<0.001	2 (2–3)	1.7 (1.3–3.0)	0.08
Length of in-hospital stay, days, (IQR)	7 (6–9)	6 (6–7)	<0.001	7 (6–9)	7 (6–8)	0.12

## Data Availability

The dataset of this study is available from the corresponding author upon reasonable request.
